# Supplementary material for: Identification of photoperiod-induced specific miRNAs in the adrenal glands of Sunite sheep (Ovis aries)
Source: Front Vet Sci. 2022 Jul 22;9:888207. doi: 10.3389/fvets.2022.888207 (PMC9354845; doi:10.3389/fvets.2022.888207)
Supplement: Supplementary file 1 [file Data_Sheet_1.ZIP › Supplementary file/miRDeep2 file.zip!/miRDeep2 file/Know_Structure/SP42A3/oar-mir-17.pdf]

miRBase precursor : oar-mir-17
Total read count : 5669
oar-miR-17-5p read count : 5629
remaining reads : 40

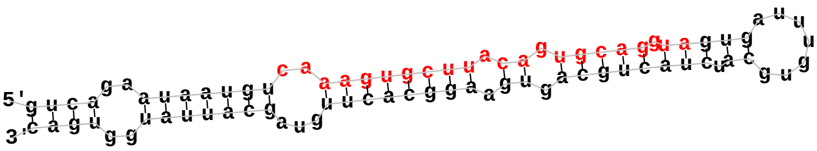

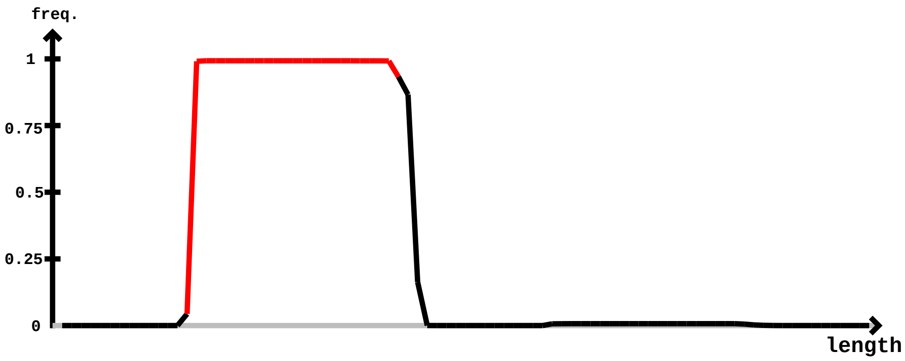

oar-miR-17-5p

5'- gucagaauaaugucaaagugcuuacagugcagguagugauuugugcaucuacugcagugaaggcacuuguagcauuauggugac -3' exp

((((..(((((((..((((((((.((.(((((.(((((.......)).)))))))).)).))))))))...)))))))..))))	reads	mm	sample
............ucaaagugcuuacagugcagg...	1	0	seq
............ucaaagugcuuacagugcaggu..	41	0	seq
............ucaaagugcuuacagugcaggua...	45	0	seq
............ucaaagugcuuacagugcagguag..	149	0	seq
............ucaaagugcuuacagugcagguagu...	12	0	seq
.............caaagugcuuacagugU..	1	1	seq
.............caaagugcuuacagugc..	1	0	seq
.............caaagugcuuacagugcaggu..	292	0	seq
.............caaagugcuCacagugcaggu..	3	1	seq
.............caaagugcuuacagugcaggua...	333	0	seq
.............caaagugcuCacagugcaggua...	1	1	seq
.............caaagugcuCacagugcagguag..	45	1	seq
.............caaagugcuuacagugcagguag..	3782	0	seq
.............caaagugcuCacagugcagguagu...	6	1	seq
.............caaagugcuuacagugcagguagu...	905	0	seq
.............caaagugcuuacagugcagguagug..	1	0	seq
..............aaagugcuuaUagugcagg...	1	1	seq
..............aaagugcuuacagugcagguag..	8	0	seq
..............aaagugcuuacagugcagguagu...	2	0	seq
....acugcagugaaggcacuugu..............	9	0	seq
....acugcagugaaggcacuugua.............	17	0	seq
....acugcagugaaggcacuuguag............	9	0	seq
....acugcagugaaggcacuuguagc...........	2	0	seq
....cugcagugaaggcacuuguagc...........	1	0	seq
....cugcagugaaggcacuuguagca..........	1	0	seq
....ugcagugaaggcacuuguagc...........	1	0	seq
